# Analysis and Quantification of the Mitochondrial–ER Lipidome

**DOI:** 10.21769/BioProtoc.5028

**Published:** 2024-07-05

**Authors:** Alexis R. Diaz-Vegas, Anthony S. Don, James G. Burchfield

**Affiliations:** 1 Charles Perkins Centre, School of life and Environmental Sciences, University of Sydney, Sydney, Australia; 2 Charles Perkins Centre and School of Medical Sciences, Faculty of Medicine and Health, University of Sydney, Sydney, Australia

**Keywords:** Mitochondria, Endoplasmic reticulum, Subcellular fractionation, Lipidomics, Ceramides, Cardiolipin

## Abstract

Mitochondria are vital organelles essential for cellular functions, but their lipid composition and response to stressors are not fully understood. Recent advancements in lipidomics reveal insights into lipid functions, especially their roles in metabolic perturbations and diseases. Previous methods have focused on the protein composition of mitochondria and mitochondrial-associated membranes. The advantage of our technique is that it combines organelle isolation with targeted lipidomics, offering new insights into the composition and dynamics of these organelles in pathological conditions. We developed a mitochondria isolation protocol for L6 myotubes, enabling lipidomics analysis of specific organelles without interference from other cellular compartments. This approach offers a unique opportunity to dissect lipid dynamics within mitochondria and their associated ER compartments under cellular stress.

Key features

• Analysis and quantification of lipids in mitochondria–ER fraction through liquid chromatography–tandem mass spectrometry-based lipidomics (LC-MS/MS lipidomics).

• LC-MS/MS lipidomics provide precise and unbiased information on the lipid composition in in vitro systems.

• LC-MS/MS lipidomics facilitates the identification of lipid signatures in mammalian cells.

## Graphical overview


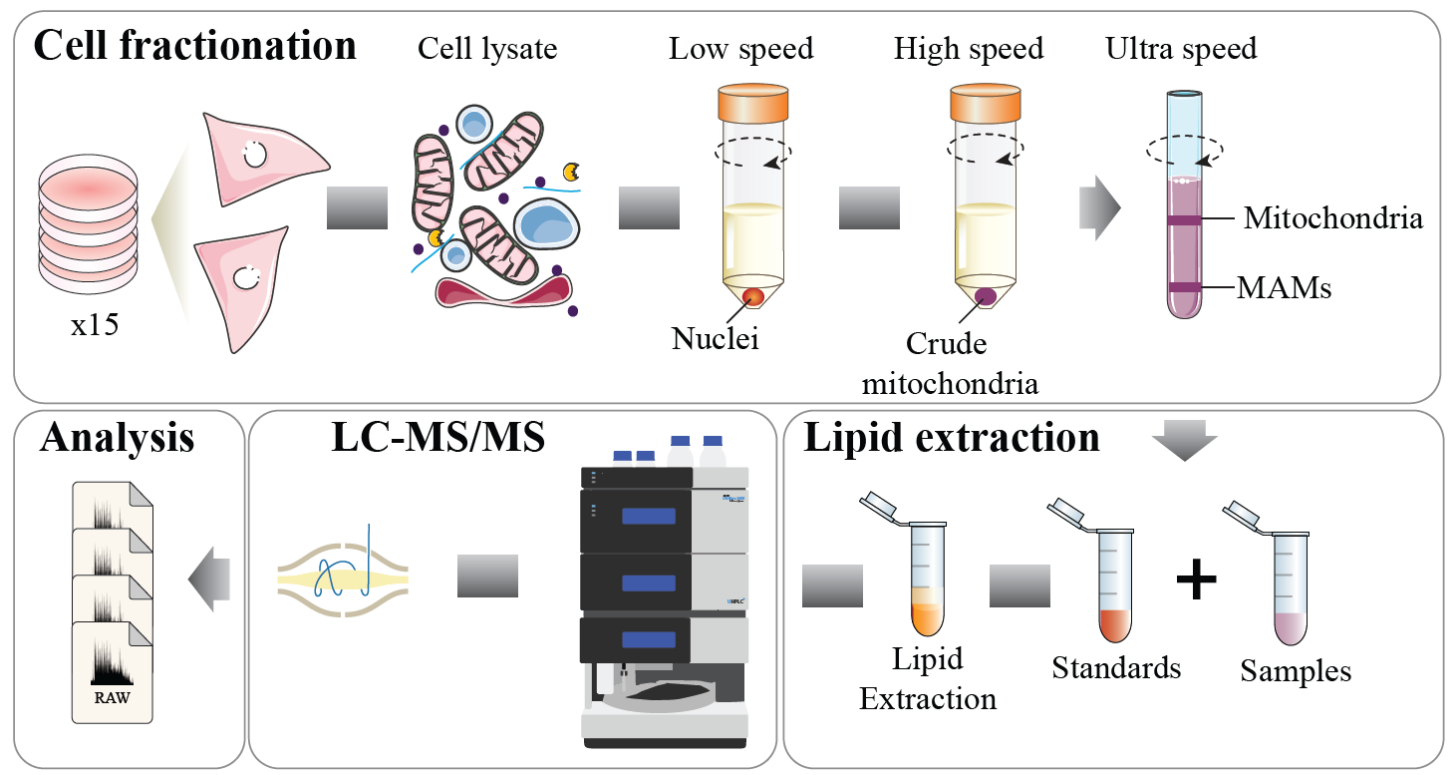


## Background

Mitochondria are essential cellular components with intricate structures and functions vital for life. Comprising two distinct membranes with unique lipid compositions, these dynamic organelles play multifaceted roles in maintaining cellular homeostasis. While many of these lipids originate from the endoplasmic reticulum (ER) and are subsequently transported to the mitochondria, the precise mechanisms governing the maintenance of mitochondrial membrane lipid composition and its response to environmental stressors remain incompletely understood.

Recent advances in detection methodologies, particularly in lipidomics, have revolutionized our understanding of lipid physiology by uncovering previously unrecognized lipid functions, such as how certain subgroups of lipid species respond to metabolic perturbations and their association with human diseases [1–3]. Although lipidomics has predominantly been applied to study changes in lipid composition at the whole tissue or cell level, less attention has been devoted to elucidating the lipid composition of subcellular compartments and how this evolves in response to cellular stress. Lipidomics analysis in subcellular fractions, such as mitochondria, enables the specific determination of lipid changes within organelles without interference from lipid signatures of other regions such as lipid droplets, nucleus, plasma membrane, and endosomal/lysosomal compartments [2,4,5].

We have implemented a mitochondria isolation protocol capable of fractionating mitochondria and mitochondria–ER fractions in the skeletal muscle cell line L6 myotubes. These fractions are then subjected to mass spectrometry (MS)-based lipidomics, enabling lipid composition dissection in these specific subcellular regions. This approach can shed light on how lipid dynamics within mitochondria and their associated ER compartments are influenced by cellular stressors [2].

## Materials and reagents

**Biological materials**

1. L6 myotubes (L6, CRL-1458, ATCC, or other suitable cell line or tissue)

**Reagents**

1. DMEM high glucose (Gibco, catalog number: A5256701)
2. Fetal bovine serum (FBS) (Gibco, catalog number: 11965092)
3. GlutaMax (Gibco, catalog number: 35050061)
4. Bovine serum albumin (BSA) (Sigma-Aldrich, catalog number: 9048-46-8)
5. Phosphate buffered saline (PBS) (Sigma-Aldrich, catalog number: P4417)
6. EGTA (Thermo, catalog number: E1219)
7. HEPES (Gibco, catalog number: 11560496)
8. Bovine serum albumin (BSA) fatty acid–free (Sigma, catalog number: A8806)
9. Protease inhibitor Complete Mini, EDTA-free (Roche, catalog number: 11836170001)
10. Mannitol (Millipore, catalog number: 63560)
11. Sodium dodecyl sulfate (SDS) (Sigma-Aldrich, catalog number: L3771)
12. Tris (VWR, catalog number: 0826)
13. Sodium chloride (NaCl) (VWR, catalog number: 27810.364)
14. Magnesium chloride (MgCl_2_) (Sigma-Aldrich, catalog number: M2670)
15. Calcium chloride (CaCl_2_) (Sigma-Aldrich, catalog number: C1016)
16. Potassium chloride (KCl) (Thermo Fisher Scientific, catalog number: AM9640G)
17. Tween-20 (Thermo Fisher Scientific, catalog number: 28320)
18. d18:1/17:0 ceramide standard (Avanti Polar Lipids, catalog number: 860517)
19. d18:1/17:0 sphingomyelin standard (Sapphire Bioscience, catalog number: 25592)
20. d17:1 sphingosine standard (Avanti Polar Lipids, catalog number: 860640)
21. d17:1 sphingosine 1-phosphate (S1P) standard (Sapphire Bioscience, catalog number: 22498)
22. 18:1/15:0 d7-diacylglycerol standard (Avanti Polar Lipids, catalog number: 791647)
23. 14:0/14:0/14:0/14:0 cardiolipin standard (Avanti Polar Lipids, catalog number: 710332)
24. Methyl-tert-butyl ether (MTBE) (Sigma-Aldrich, catalog number: 650560)
25. Methanol (Sigma-Aldrich, catalog number: 34860)
26. Water HPLC grade (Sigma-Aldrich, catalog number: WX0004)
27. Formic acid (Merk, catalog number: 1.00264)
28. Ammonium formate (Merk, catalog number: 70221)
29. Acetonitrile (Sigma-Aldrich, catalog number: 34851)
30. 2-propanol (Sigma-Aldrich, catalog number: 270490)
31. Percoll (Thermo Fisher, Catalog number: B22095.09)

**Solutions**

1. Cell culture media (see Recipes)
2. Basal media (see Recipes)
3. DPBS (see Recipes)
4. Stock solutions (see Recipes)
5. Lysis buffer 1 (see Recipes)
6. Percoll gradient (see Recipes)
7. Lysis buffer 2 (see Recipes)
8. Sample buffer (see Recipes)
9. Mobile phase A (see Recipes)
10. Mobile phase B (see Recipes)
11. Internal standard (see Recipes)

**Recipes**

1. **Cell culture media**
  DMEM high glucose
  1 mM GlutaMax
  10% FBS
  For 500 mL of DMEM high glucose, add 5.5 mL of Glutamax and 50 mL of FBS.
2. **Basal media**
  DMEM high glucose
  1 mM GlutaMax
  0.2% BSA with fatty acid
  For 500 mL of DMEM high glucose, add 5.5 mL of Glutamax and 0.2 g of BSA.
3. **DPBS**
  PBS
  1 mM MgCl_2_
  1 mM CaCl_2_
  For 100 mL of PBS, add 1 mL of MgCl_2 _(stock 1 M) and 1 mL of CaCl_2 _(stock 1 M).
4. **Lysis buffer 1**
  250 mM Mannitol
  5 mM HEPES pH 7.4
  0.5 mM EGTA (see Note 1)
  0.1% BSA with fatty acid
  Adjust pH to 7.4 with KOH
  On the day of use: 1/100 volume of protease inhibitor Complete Mini.
  This buffer has to be prepared fresh from stock solutions, and powder BSA needs to be added at the end.
  Keep this buffer on ice throughout the whole protocol.
5. **Percoll gradient**
  250 mM mannitol
  5 mM HEPES pH 7.4
  0.5 mM EGTA [ethylene glycol-bis(β-aminoethyl ether)-N,N,N′,N′-tetraacetic acid], also known as egtazic acid (INN, USAN)
  0.1% BSA with fatty acid
  18% Percoll
  Use Lysis buffer 1 (see Note 6) to dilute Percoll to the desired concentration.
6. **Lysis buffer 2**
  250 mM mannitol
  5 mM HEPES pH 7.4
  0.5 mM EGTA
  Adjust pH to 7.4 with KOH
  On the day of use: 1/100 volume of protease inhibitor cocktail set III
  This buffer has to be prepared fresh from stock solutions.
  Keep this buffer on ice throughout the whole protocol.
7. **Lipid extraction solvent**
  For lipid extraction, each solvent is added individually to the homogenate (see methods) with a ratio of 10:3:2.5 (v:v:v) of methyl-tert-butyl ether (MTBE):Methanol:Water
8. **Resuspension solvent**
  80% MeOH
  20% deionized water
  0.2% formic acid
  2 mM ammonium formate
9. **LC-MS/MS mobile phase A**
  0.1% formic acid
  10 mM ammonium formate
  60% acetonitrile
  40% water
10. **LC-MS/MS mobile phase B**
  0.1% formic acid
  10 mM ammonium formate
  90% 2-propanol
  10% acetonitrile
11. **Internal standard**
  50 µL is added to each sample
  40 µM d18:1/17:0 sphingomyelin (2 nmol/sample)
  10 µM d18:1/17:0 ceramide (0.5 nmol/sample)
  4 µM d17:1 sphingosine (0.2 nmol/sample)
  4 µM d17:1 sphingosine 1-phosphate (0.2 nmol/sample)
  40 µM 18:1/15:0 d7-diacylglycerol (2 nmol/sample)
  80 µM 14:0/14:0/14:0/14:0 cardiolipin (4 nmol/sample)
  100 µM d7-cholesterol (5 nmol/sample)
  Additional internal standards may be added for the measurement of other lipid classes.
  The internal standard mixture can be stored at -30 °C.

**Laboratory supplies**

1. 50 mL conical tube (Corning, catalog number: 001634)
2. Polypropylene tube, 14 mL, 14 mm × 95 mm (Beckman Coulter, catalog number: 331374)
3. 2 mL tubes, 13 mm × 25 mm (Beckman Coulter, catalog number: 357329)
4. Micro tube 3810X, 1.5 mL (Eppendorf, catalog number: EPPE0030125.150)
5. Safe-lock tubes 2.0 mL (Eppendorf, catalog number: 0030120.094)
6. 1 mL syringe
7. 5 mL glass tubes
8. HPLC tubes
9. Corning^®^ tissue-culture treated culture dishes (Corning, catalog number: CLS4305990)
10. Micropipettes

## Equipment

1. Refrigerated microcentrifuge
2. 2.1 100 mm Waters Acquity UPLC C18 column (1.7 μm pore size) (Thermo, model: 186002352)
3. Sonicator water bath (Thermoline, model: 505)
4. Savant^TM^ SpeedVac^TM^ high-capacity concentrators (Thermo Fisher, catalog number: SC210A-230)
5. TSQ Altis triple quadrupole mass spectrometer coupled to a Vanquish HPLC system (Thermo Fisher Scientific, model: TSQ03-11002)
6. Cell homogenizer with 18 μm ball (ISOBIOTEC)
7. Thermo centrifuge, ultra-speed (Thermo Fisher, model: WX100, catalog number: 096-247028)
8. TLA-110 fixed-angle rotor (Beckman Coulter, catalog number: 366735)
9. SW 40 Ti swinging-bucket rotor (Beckman Coulter, catalog number: 331301)
10. JA-12 fixed-angle aluminum rotor (Beckman Coulter, catalog number: 360992)
11. Precision balance FZ-i Series 320 g × 0.001 g (Australian Scientific, catalog number: FZ-300i)
12. Electrophoresis chamber (Life Technologies, catalog number: EI0002)
13. Transfer apparatus (Life Technologies, catalog number: EI0002)
14. Sponge pads for XCell II blotting (Life Technologies, catalog number: EI9052)
15. Cell scraper
16. Odyssey^®^ DLx imaging system (LI-COR, model: BX41-PH-B)
17. Vortex mixer (Ratek, model: VM1)

## Software and datasets

1. Image Studio^TM^ (LI-COR)
2. TraceFinder software (Thermo Fisher)
3. Microsoft Excel

## Procedure

We describe below the step-by-step procedure for performing a mitochondria–ER isolation followed by lipidomic analysis and quantification [6]. This procedure has been applied to both L6 myotubes and HeLa cells and published in Diaz-Vegas et al. [2]. For a different cell line, further optimization might be necessary (e.g., starting material). Store all buffers at 4 °C and perform the procedure on ice. Room temperature is defined as 22 °C throughout this protocol. Washing steps are provided for each step throughout the protocol. For this protocol, 15 fully confluent dishes of 15 cm were pulled together to obtain enough material to isolate the different fractions (equal to one biological replicate).

- **Mitochondria–ER isolation**
  1. Before starting
    - Cool cell homogenizer to 4 °C.
    - Cool down DPBS.
    - Cool down rotors (TLA110, sw40Ti, JA-12).
    - Cool down centrifuges.
    - Cool down 50 and 1.5 mL tubes.
  2. Basal period
    - Cultivate L6 cells to 100% confluency in 15 cm diameter tissue culture plates (15 dishes per condition).
    - Remove the media and wash cells with room-temperature PBS (three times, 6 mL each time).
    - Add 10 mL of basal media and incubate at 37 °C for 2 h in the incubator (95% O_2_, 5% CO_2_).
  3. Harvesting and cell lysis
    - Transfer the dishes to an ice tray.
    - Immediately, wash the cells five times with ice-cold DPBS by gently adding the buffer against the wall of the dish to prevent cell loss.
    - After the last wash, add 4 mL of lysis buffer 1 to one plate.
    - Using a cell scraper, remove the cells (see Note 2).
    - Tilt the plate to allow cells to accumulate in one region. When the first plate is ready, remove DPBS from a second plate and transfer the entire volume from plate 1 to plate 2.
    - Repeat the scrapping process in the new dish. Continue this procedure until all plates have been scraped, collecting the entire sample (see Note 3).
    - Transfer the collected sample to an ice-cold 50 mL conical tube.
    - Spin down cells at 1,500 rpm (300× *g*) for 5 min in a JA-12 rotor at 4 °C.
    - Discard supernatant and resuspend cells in 5 mL of lysis buffer 1.
    - Assemble the cell homogenizer (see Note 4).
    - Equilibrate the cell homogenizer with 1 mL of lysis buffer 1. Discard this buffer after equilibration.
    - Transfer cell suspension using a 1 mL syringe and process the samples using the cell homogenizer.
    - Pass 10 times back and forth through the cell homogenizer (see Note 5).
    - Transfer lysate to a new ice-cold 50 mL Falcon tube. Repeat step A3m until all samples are collected in this new Falcon tube (see Note 6).
    - Spin down nuclei for 10 min at 1,810 rpm (600× *g* at rmax) in a JA-12 rotor at 4 °C.
    - Transfer supernatant (~4 mL) to a new ice-cold 50 mL Falcon tube and discard pellet.
    - Divide the supernatant into two ice-cold 1.5 mL Eppi^®^ tubes for isolation of crude mitochondria (reserve 1 mL as whole-cell lysate for further experiments, e.g., lipidomics in whole lysate, western blotting, etc., and store at -80 °C).
  4. Crude mitochondria isolation
    - Transfer the 1.5 mL Eppi^®^ tubes to a refrigerated microcentrifuge and spin at 8,500 rpm (10,300× *g* at rmax) for 10 min in a JA-12 rotor at 4 °C. This will separate crude mitochondria (pellet) from post-mitochondrial fraction (supernatant).
    - Transfer supernatant containing post-mitochondrial fraction to a new 1.5 mL Eppi^®^ tubes and store at -80 °C.
    - Resuspend pellet in 1 mL of lysis buffer 1 by gently pipetting up and down (see Note 7).
  5. Isolation of mitochondria–ER fractions
    - Carefully, layer the suspension into a polycarbonate tube containing 7.9 mL of 18% Percoll gradient (see Note 8). Add 3 mL of Lysis buffer 1 on top with a p1000 to calibrate the tubes (see Note 9).
    - Weigh each polycarbonate tube and balance with Lysis buffer 1 (± 0.001 g) (Note 10).
    - Centrifuge at 95,000× *g* at rmax for 30 min at 4 °C using a sw40Ti rotor.
    - With a 1 mL syringe, aspirate the band at the top portion of the tube and transfer it (~2 mL) into two Beckman 1.4 mL tubes (1 mL per tube). This first band will contain the mitochondria–ER fraction.
    - With another 1 mL syringe, aspirate the band at the bottom of the tube and transfer it (~2 mL) into four Eppi^®^ tubes (500 µL per tube). This will contain the pure mitochondrial fraction.
    - Weigh each Beckman 1.4 mL tube and balance with Lysis buffer 1 (± 0.001 g).
    - Spin down the mitochondrial-associated membranes (MAM) fraction at 60,000 rpm for 1 h using the TLA 110 rotor at 4 °C.
    - Mitochondria–ER will be a mucous pellet on a clear Percoll sediment at the bottom of the tube. Aspirate out Mitochondria–ER carefully with a micropipette and transfer it to an Eppi^®^ tube (~200 µL) (see Note 11).
    - Wash the mitochondria–ER pellet three times with lysis buffer 2 (max speed for 15 min in JA-12 rotor at 4 °C).
    - Add 50 µL of 5× sample buffer and adjust the volume to 300 µL with 1× sample buffer for western blotting or store at -80 °C for lipid extraction.
    - Add 1 mL of lysis buffer 1 to each tube with pure mitochondria and mix (see Note 7).
    - Spin down the mitochondrial fraction at 10,000 rcf for 10 min at 4 °C.
    - Mitochondria will form a loose pellet. Carefully aspirate most of the supernatant (~1.2 mL) and replace it with more lysis buffer 1.
    - Spin down the mitochondrial fraction at 10,000 rcf for 10 min at 4 °C.
    - Aspirate supernatant and resuspend pellet in 300 µL of 1× sample buffer (100 µL per tube) or store at -80 °C for lipid extraction. Resuspension requires repeated up and down pipetting.
    Analysis of protein content is determined by the BCA method. In these fractions, cytochrome content can be evaluated using the method of Vanneste [7] and Nicholls [8]. These mitochondrial preparations can be assayed for CoQ content by LC-MS/MS after extraction [9].
- **Lipid extraction and lipidomics analysis**
  1. Quality control samples
    - Blank solution without internal standards.
    - Blank solution with internal standards (see Recipes for internal standards).
    - Set up pooled quality control samples (replicates) to be observed for variations throughout the analytical run. This may involve preparing a whole-cell lysate. For this experiment, we used a pool sample from all conditions as the control.
  2. Lipid extraction
    This procedure was adapted from Matyash et al. [10].
    - Prepare internal standard mixture containing 2 nmol d18:1/17:0 sphingomyelin, 0.5 nmol d18:1/17:0 ceramide, 0.2 nmol d17:1 sphingosine, 0.2 nmol d17:1 sphingosine-1-phosphate (S1P), 2 nmol 18:1/15:0 d7-diacylglycerol, 2 nmol 14:0/14:0/14:0/14:0 cardiolipin, and 5 nmol d7-cholesterol per sample, diluted in 50 µL of methanol per sample. Additional internal standards may be included for other lipid classes (see Note 12).
    - Use 30 μg of mitochondrial protein for extraction.
    - Add 200 μL of MeOH (MS grade) and 50 μL of internal standard stock to safe-lock tubes (2.0 mL) (see Note 13).
    - Add 850 μL of MTBE to the tube and sonicate in the bath sonicator in the cold room for 30 min. Check every 10 min to ensure the bath remains cold (see Note 15).
    - Add 212 μL of MilliQ water to each tube to induce phase separation (see Notes 15–16).
    - Vortex the sample at max speed for 10 sec and spin at 2,000× *g* for 5 min to complete phase separation.
    - Transfer the upper organic phase into 5 mL glass tubes (see Note 15).
    - Repeat steps B2b–f twice to re-extract the lower phase. Add 2 mL of the solvent mixture, whose composition is equivalent to the expected composition of the upper phase (obtained by mixing MTBE/methanol/water 10:3:2.5, v/v/v) for each re-extraction (see Note 15).
    - Collect the upper phases and combine them in the 5 mL glass tube.
    - Dry the extract overnight in a Speedvac under a low-heat setting.
    - Reconstitute the extract in 400 μL of 0.1% FA/1 mM ammonium formate in 80% MeOH (cover the sample vials with parafilm). Vortex for 10 s at max speed at room temperature (see Note 16).
    - Centrifuge the 5 mL glass tubes carefully at 2,000× *g* for 10 min to pellet insoluble material, and then transfer 300 μL of the methanol extract to a glass HPLC vial.
    - Store vials at -30 °C for further analysis.
  3. Lipid quantification by targeted lipidomics
    - Lipids are separated on a 2.1 × 100 mm Waters Acquity UPLC C18 column (1.7 μm pore size) using a flow rate of 0.28 mL/min.
    - Total run time is 25 min with a binary solvent gradient, starting at 20% of mobile phase B (80% A) and holding for 3 min, ramping up to 100% B from 3 to 14 min, holding at 100% B from 14 to 20 min, returning to 20% B at 20.5 min, and holding at 20% B for a further 4.5 min.
    - Lipids are detected on a triple quadrupole or quadrupole-ion trap instrument operating in positive ion mode. Each lipid species is detected as a distinct precursor-product ion pair using selected ion monitoring.
    - Ceramides, sphingomyelin, sphingosine, and sphingosine 1-phosphate are identified as the [M+H]+ precursor ion, with *m/z* 262.3 (sphinganine), 264.3 (sphingosine), or 266.3 (sphinganine) product ion, and *m/z* 184.1 product ion in the case of sphingomyelin. Diacylglycerols (DAGs) are identified as the [M + NH_4_]+ precursor ion and product ion corresponding to neutral loss of a fatty acid. Cardiolipins are identified as the [M + H]+ precursor ion and product ion corresponding to a constituent DAG [M + H - H_2_O]+ ion. Cholesterol is detected using precursor m/z 369.4 and product m/z 161.1.
    - Quantification of lipid profile in whole lysate and mitochondrial fraction is shown in Figure 1.
  **Figure 1.**
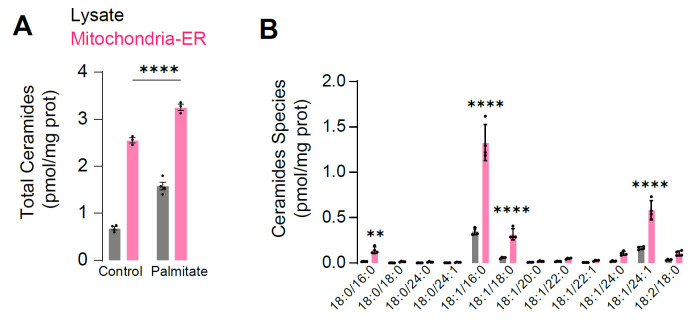
Representative results from targeted lipidomic for ceramides. A. L6 myotubes were incubated with the fatty acid palmitate (150 µM for 16 h) and ceramide levels were measured in whole lysate or mitochondrial–ER fraction. B. Abundance of the different ceramide species in lysate and mitochondrial–ER fraction. N = 3–4 biological replicates, **p < 0.001, ****p < 0.0001.

## Data analysis

Lipids are quantified as the area under the peak for a specific precursor–product ion pair. It is recommended that each peak comprises a minimum of 8–10 scans for that specific precursor–product ion pair. Peak detection and integration is performed with vendor-specific software or freeware such as Skyline [11]. For our analyses, TraceFinder was used. The amount of each lipid is determined first as the ratio to its class-specific internal standard. This is then multiplied by the amount of internal standard added to estimate the nmol of each lipid in the sample, then divided by the amount of protein used for lipid extraction, so that lipid levels are expressed as nmol lipid/mg protein. Levels of individual lipid species may then be compared between sample groups or treatment conditions using statistical analyses appropriate to the experimental design.

## Validation of protocol

This protocol has been used and validated in the following research article(s):

- Diaz-Vegas et al. [2], Mitochondrial electron transport chain, ceramide, and coenzyme Q are linked in a pathway that drives insulin resistance in skeletal muscle, eLife 12, RP87340 (Figure 2, panel C, D; Figure 3, panel C, D).
  Ceramide species were quantified using targeted lipidomics. N = 3 biological replicates. Control conditions refer to cells with vehicle control for each experiment. Student’s *t*-test was utilized for comparing two groups, whilst ordinary one-way ANOVA followed by Dunnett’s multiple-comparison test was employed for comparing multiple groups.

## General notes and troubleshooting

**General notes**

1. The use of EGTA is essential to avoid mitochondrial calcium overload during isolation.
2. Keep the other 14 dishes on ice with 10 mL of DPBS during this process.
3. Volume will rise to ~12 mL.
4. It is possible that different ball sizes are necessary for different cell lines. In this protocol, we used an 18 μm ball size for cell lysis.
5. It is critical to keep consistency in speed to obtain consistent results without damaging mitochondria structure.
6. Wash and dry the homogenizer after the whole 5 mL of sample has been passed (once with tap water and then with distilled water).
7. Use a 1 mL blunt tip to prevent mechanical mitochondrial damage during this step.
8. Use a 1 mL syringe for better results.
9. This volume can vary depending on the polycarbonate tube size.
10. Dry out the surface of the polycarbonate tube; otherwise, it will stick to the centrifuge and it will be almost impossible to take it out.
11. Do not aspirate the supernatant, as mitochondria–ER pellet is loose.
12. Prior to extraction, thaw internal standards to room temperature for 1 h and then vortex.
13. Additional internal standards should be included if you wish to measure other lipid classes. Internationally accepted conventions (Lipidomics Standards Initiative, https://lipidomicstandards.org/) recommend the inclusion of at least one internal standard for each lipid class of interest. See for example [12]. You may use deuterated internal standards for sphingolipids, in place of the d17:1 and d18:1/17:0 variants.
14. Place a mixture of water and ice inside the sonicator to ensure samples are cold during this step. Remove excess liquid and add ice if multiple runs of sonication are required.
15. MTBE, when mixed with air, can cause a hazardous/flammable atmosphere. Always use this chemical in a fume hood.
16. Subtract from this 212 μL of the volume associated with the sample. For instance, if the 30 μg of mitochondrial proteins were in 50 μL of buffer, here we only need to add 162 μL of MilliQ (212 − 50 = 162). This is critical to maintain the MeOH:MTBE:H_2_O ratio.
17. Thoroughly wrap each tube with parafilm before vortexing.
